# Nature-Inspired Effects of Naturally Occurring Trace Element-Doped Hydroxyapatite Combined with Surface Interactions of Mineral-Apatite Single Crystals on Human Fibroblast Behavior †

**DOI:** 10.3390/ijms23020802

**Published:** 2022-01-12

**Authors:** Malgorzata Tyszka-Czochara, Marzena Suder, Agnieszka Dołhańczuk-Śródka, Małgorzata Rajfur, Katarzyna Grata, Michał Starosta, Agnieszka Jagoda-Pasternak, Wiktor Kasprzyk, Anna K. Nowak, Saeid Ahmadzadeh, Dorota Kopeć, Piotr Suryło, Tomasz Świergosz, Katarzyna M. Stadnicka

**Affiliations:** 1 Faculty of Pharmacy, Jagiellonian University Medical College, Medyczna 9, 30-688 Krakow, Poland; 2 Department of Crystal Chemistry and Crystal Physics, Faculty of Chemistry, Jagiellonian University, Gronostajowa 2, 30-387 Krakow, Poland; sudermz@gmail.com (M.S.); katarzyna.stadnicka@uj.edu.pl (K.M.S.); 3 Institute of Environmental Engineering and Biotechnology, University of Opole, Kominka 6, 45-035 Opole, Poland; agna@uni.opole.pl (A.D.-Ś.); rajfur@uni.opole.pl (M.R.); kgrata@uni.opole.pl (K.G.); mstarosta.93@o2.pl (M.S.); 4 Department of General and Inorganic Chemistry, Faculty of Chemical Engineering and Technology, Cracow University of Technology, Warszawska 24, 31-155 Krakow, Poland; agnieszka.jagoda-pasternak@pk.edu.pl (A.J.-P.); anna.k.nowak@pk.edu.pl (A.K.N.); 5 Department of Biotechnology and Physical Chemistry, Faculty of Chemical Engineering and Technology, Cracow University of Technology, Warszawska 24, 31-155 Krakow, Poland; wiktor.kasprzyk@pk.edu.pl; 6 Pharmaceutics Research Centre, Institute of Neuropharmacology, Kerman University of Medical Sciences, Kerman 7616913555, Iran; chem_ahmadzadeh@yahoo.com; 7 Pharmaceutical Sciences and Cosmetic Products Research Centre, Kerman University of Medical Sciences, Kerman 7616913555, Iran; 8 Department of Chemical Technology and Environmental Analysis, Faculty of Chemical Engineering and Technology, Cracow University of Technology, Warszawska 24, 31-155 Krakow, Poland; dorota.kopec@pk.edu.pl (D.K.); piotr.surylo@pk.edu.pl (P.S.)

**Keywords:** hydroxyapatite, mineral apatite single crystals, FTIR, SEM-EDXS, X-ray diffraction, fibroblast cell culture, cell–surface interactions

## Abstract

Innovative engineering design for biologically active hydroxyapatites requires enhancing both mechanical and physical properties, along with biocompatibility, by doping with appropriate chemical elements. Herein, the purpose of this investigation was to evaluate and elucidate the model of naturally occurring hydroxyapatite and the effects of doped trace elements on the function of normal human fibroblasts, representing the main cells of connective tissues. The substrates applied (geological apatites with hexagonal prismatic crystal habit originated from Slyudyanka, Lake Baikal, Russia (GAp) and from Imilchil, The Atlas Mountains, Morocco (YAp)) were prepared from mineral natural apatite with a chemical composition consistent with the building blocks of enamel and enriched with a significant F^−^ content. Materials in the form of powders, extracts and single-crystal plates have been investigated. Moreover, the effects on the function of fibroblasts cultured on the analyzed surfaces in the form of changes in metabolic activity, proliferation and cell morphology were evaluated. Apatite plates were also evaluated for cytotoxicity and immune cell activation capacity. The results suggest that a moderate amount of F^−^ has a positive effect on cell proliferation, whereas an inhibitory effect was attributed to the Cl^−^ concentration. It was found that for (100) GAp plate, fibroblast proliferation was significantly increased, whereas for (001) YAp plate, it was significantly reduced, with no cytotoxic effect and no immune response from macrophages exposed to these materials. The study of the interaction of fibroblasts with apatite crystal surfaces provides a characterization relevant to medical applications and may contribute to the design of biomaterials suitable for medical applications and the evaluation of their bioavailability.

## 1. Introduction

Hydroxyapatite (HAp) substitutions have the ability to induce both positive and negative effects on cell viability but also on the mechanical properties and solubility of biomaterials [1,2,3,4]. Synthetic equivalents of HAp have high biocompatibility and are commonly employed in clinical practice [5,6,7]. 

Due to its structural similarity to biological bone, visible biocompatibility, and bioactivity, HAp has been applied in medicine and dentistry in several forms: as dense sintered ceramics; porous forms; granules; and coatings of metal implants [8,9,10]. The shape of HAp crystallites is highly anisotropic, with at least two types of crystal faces: (100) and (101), and rarely (001), exhibiting different distributions of electrostatic potential, different interactions with water, and different adsorptions of biomolecules [11]. Therefore, surface charges measured on individual grains and across grain boundaries of polycrystalline HAp vary in terms of nanoscale and depend on preferentially exposed crystal faces [12]. It has been shown that HAp ceramics with controlled orientation can alter material bioactivity and cell adhesion, in addition to their performance [13,14]. Moreover, for HAp-coated implant surfaces, the response of living cells to the exposed orientation should also be considered, as HAp crystallites may have a preferred orientation, e.g., with (001) parallel to the surface of the coating, as was observed for HAp-coated titanium [15]. 

In the last decade, contrary to mineralogical sciences, the research in the field of biomaterials has focused on nano-sized apatite crystals with defined a chemical composition, spatial orientation, and morphology [16], which could comprise a part of composites mimicking bone structure both in the aspects of mechanical properties and biological response [17,18]. However, even in such systems the crystallographic orientation of the crystallites appeared to be as significant as it is for their mineral monocrystalline counterparts. The morphology and the particle size of HAp crystallites could be controlled during synthesis by adjusting certain additives, such as anionic polymers having strong affinity to Ca^2+^, and thus adsorbing preferentially onto (100) faces of the formed HAp [19]. The HAp nanocrystals, as a part of biomimetic composites, grown in vitro through interactions with biomolecules, such as atelocollagen or recombinant human-like collagen, were shown to exhibit a preferential orientation [20,21].

All these results indicate that the orientation of HAp crystallites is one of the most significant parameters affecting the interaction between living cells and biomaterial surface. Considering the implantation of synthetic HAp material, one should take into account that the cells of adjacent tissue have direct contact with the periodic surface of crystallites with particular arrangement of Ca^2+^, PO_4_^3−^ and OH^−^ ions instead of the hierarchical bone structure. The question arises as to what extent the connective tissue cells (like fibroblasts) could recognize the structure of substratum (such as apatite) at atomic level.

Additionally, the improvement of the physical and mechanical properties of the materials, combined with good biocompatibility, can be achieved using a trace number of specific dopants in synthetic HAp [22,23]. Recently, various ions substituted in calcium phosphate apatite, such as Na^+^, K^+^, Mg^2+^, Sr^2+^, Zn^2+^, CO_3_^2−^, SiO_4_^4−^, F^−^ and Cl^−^, were shown to affect cell response [24,25,26,27,28,29,30]. In particular, carbonated Na, Mg, K, F and Cl-substituted HAp had a stimulating effect on transformed MC3T3-E1 cells [30]; however, CO_3_^2−^ decreased the mechanical properties of the biomaterial. Some ions, such as Mg^2+^ and SiO_4_^4−^, were found to promote osteoblast-like cell proliferation and differentiation [26,27]. F^−^ ions were shown to stabilize hydroxyapatite structure and decrease solubility, and were proved to be an important additive that may increase the bond strength of HAp coatings onto Ti dental implants [31]. Much less attention was paid to the biological effects of F^−^. Cheng et al. [32] reported the increase of attachment and proliferation of cells grown on fluorhydroxyapatite. The biological effect of small additions of other ions, such as SO_4_^2−^ and Cl^−^ [24], is not fully understood yet. 

The studies conducted so far have been mainly focused on the interactions between osteosarcomas and the HAp surface. However, there is much less data concerning the influence of substituted HAp on normal connective tissue cells (fibroblasts). The influence may be important, especially in the aspect of biomaterial engineering for biomedical applications, since the connective tissue cells are a main line of interaction between implanted material and living tissue [33,34,35,36]. Therefore, normal human fibroblasts, which in vivo are adjacent both to hard tissues and to HAp implants, were used in our experiments. The main objective of our study was to investigate cell interactions with the surface of solid, naturally occurring apatite biomaterials, which contain selected ionic substitutions. The single crystals of mineral apatite were used as the substrates for cell culture growth. The apatites were of optimal chemical composition, containing trace dopants of biologically relevant ions, and were applied in the form of single-crystal slabs exposing particular crystallographic planes (‘apatite plates’). In line with the assumed model, the studies concerning apatite biocompatibility were supplemented with the data on cytotoxicity and the immune cell response. Using the oriented apatite plates, the cell culture study aimed to: (i) estimate if human fibroblasts are able to grow and proliferate on mineral surfaces; (ii) elucidate if cell growth depends on apatite-plate crystallographic orientation and how the cell growth changes during incubation; (iii) determine if the plates may induce detrimental cytotoxic effect on the cells; and (iv) evaluate if the molecular mechanism of cell injury involves apoptosis and/or necrosis processes. 

## 2. Results

Prior to cell culture experiments, physical and chemical analyses of the substrate materials GAp, YAp and HAp were described. Culture of fibroblasts on apatite plates included cell proliferation assays, LDH release assay, quantification of apoptosis and necrosis, changes in cell morphology, and assessment of cell adhesion strength.

### 2.1. Mineral Apatites 

#### 2.1.1. Surface and Bulk Characteristics of the Plates

**Figure A1:**
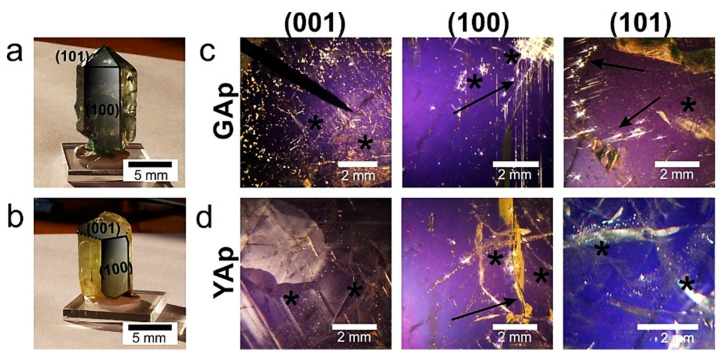
The single crystals of natural mineral apatites GAp (**a**) and YAp (**b**), with well-developed crystal faces corresponding to the (100) and (101) faces marked with continuous lines. The (001) cut of the crystal, shown in (**b**), is marked with broken lines. (**c**) The GAp (001), (100) and (101) plates are shown in polarized light. The inclusions are seen as illuminated irregularly-shaped points of variable diameters. The accumulations of tiny (‘foggy’) inclusions and the longitudinal-shaped ones (paralleled to c-axis) are marked with asterisks and arrows, respectively. The YAp plates are shown in polarized light (**d**). The inclusions are seen as bright, bubble-like points arranged in bands (marked with asterisks). The arrow indicates fracture in the fragment of (100) plate.

The mineral apatite crystals and the apatite plates, used for experiments with cells, are shown in Figure A1 (Appendix A). The observations under polarizing microscope revealed that the apatite plates were transparent, almost homogenous; nevertheless, typically for mineral natural samples, they contained several types of inclusions (solid, liquid, and gaseous ones), usually of longitudinal shape and arranged parallel to the hexagonal axis of the crystal. 

**Figure A2:**
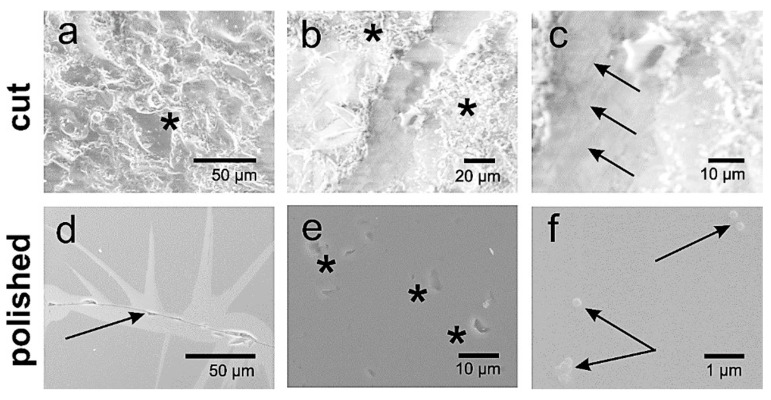
The SEM micrographs of the fresh cut GAp (**a**–**c**) and finely polished YAp (**d**–**f**) surfaces. (**a**) Diamond-saw cut surface of GAp (001) plate with smooth cleaved fragment marked by an asterisk. (**b**) GAp (100) plate with fractured areas (marked by asterisks) and smooth fragment in between. (**c**) The higher magnification (1000×) of (**b**) stepped cleavage of smooth fragment–arrows marks the edges of steps. (**d**) Inclusion or strain area in YAp (101) plate surrounding a fracture pointed with the arrow. (**e**) The fragment of (101) YAp plate with post-inclusion cavities (marked with the asterisks). (**f**) The higher magnification (×10,000) of finely polished YAp surface with the bubble-like inclusions or debris indicated by the arrows.

Some examples of variable-pressure SEM micrographs, obtained for the freshly cut plates of the apatite GAp, were shown in Figure A2a–c (Appendix A), and those obtained for the polished plates of the apatite YAp are shown in Figure A2d–f (Appendix A). The micrographs were chosen to indicate differences between the freshly cut and the polished plates and to prove smoothness of the surfaces (see Figure A2f, Appendix A) used for the cell culture. Additionally, the polishing procedure was standardized to eliminate possible differences in submicron topography between plates, which might influence cell culture experiments. Isolated inclusions or debris (Figure A2d,f, Appendix A) were in general in the bulk of the minerals. At the surface of apatite plates only occasional empty cavities were left (compare Figure A2e, Appendix A), and in most cases they were removed during polishing. 

The chemical compositions of the apatites were assessed by SEM-EDXS detector, and the results are summarized in Table 1. There were no significant differences of chemical content for the plates of different crystallographic orientation (cut from various fragments of the specimens) within particular mineral type (GAp or YAp). Both minerals were fluorhydroxyapatite, but those that originated from Imilchil (YAp) had chloride ions beside the fluoride and hydroxyl ions. The mineral apatites, with general chemical formulae Ca_10_(PO_4_)_6_(F,Cl,OH)_2_, had trace amounts of other chemical elements such as Si and S in the form of SiO_4_^4−^ and SO_4_^2−^ groups substituting PO_4_^3−^, and Na substituting Ca (as cations). The averaged chemical compositions with charge compensated by HPO_4_^2−^ were as follows: 

[Ca_9.06_Na_0.10_]_∑ = 9.16_ [(PO_4_)_3.86_(HPO_4_)_1.82_(SO_4_)_0.17_(SiO_4_)_0.16_]_∑ = 6.01_ [F_1.62_(OH)_0.38_]_∑ = 2.00_ for GAp and Ca_9.60_ [(PO_4_)_5.00_(HPO_4_)_0.90_(SiO_4_)_0.10_]_∑ = 6.00_ [F_1.17_Cl_0.17_(OH)_0.66_]_∑ = 2.00_ for YAp.

The molar concentrations of those common substitutions correlate with each other and hence are probably consistent with co-substitution charge-compensation mechanisms broadly described in the literature [37]. However, relatively low Ca^2+^ contents suggest the presence of carbonate group in the crystal structure according to the equation proposed in [37].

**Figure A3:**
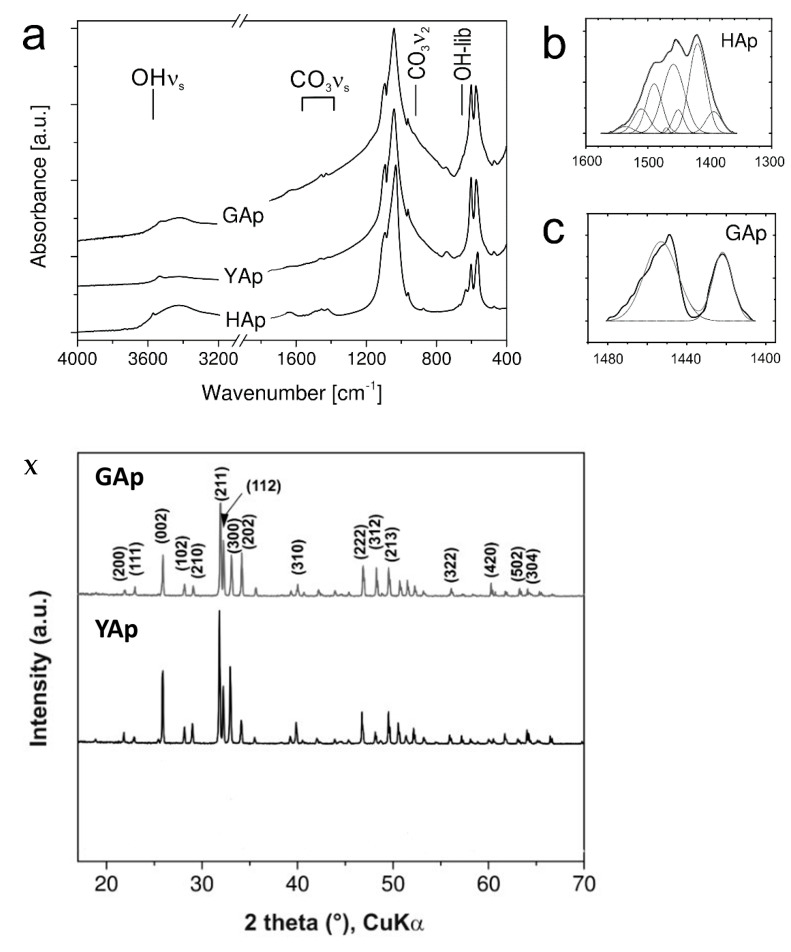

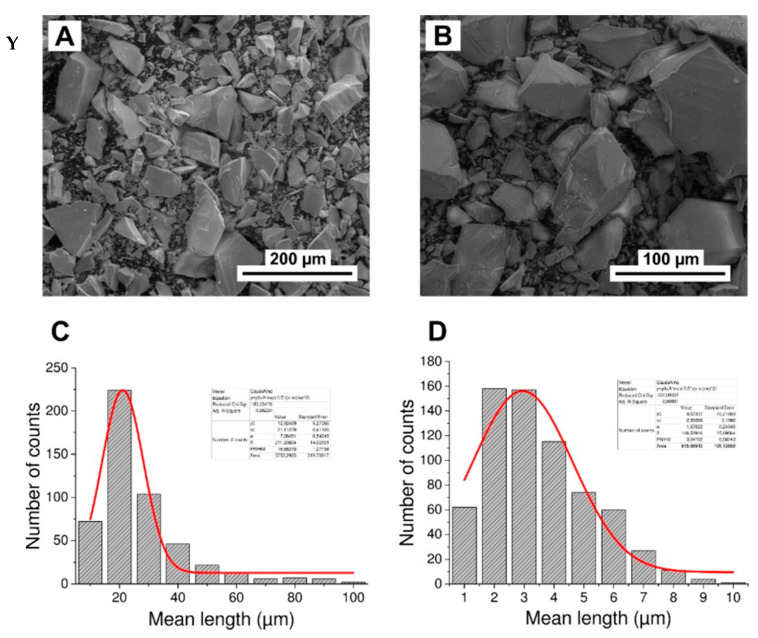
The FTIR spectra for GAp, YAp and HAp (nanoXIM HAp403) powders (**a**). The regions of stretching vibration of hydroxyl groups (OH ν_s_), of carbonate ions (CO_3_ ν_s_), and of hydroxyl group libration bands (OH-lib) are marked. Asymmetric stretch (ν_3_) regions of the FTIR spectrum fitted with Gaussian distributions corresponding to the peaks and shoulders evident in envelope are shown for HAp (**b**) and GAp (**c**). **X:** Powder X-diffraction lines identified by Miller indices (hkl) for lattice planes. **Y:** SEM micrographs of GAp powder (**A**,**B**). LVSED (low-vacuum secondary electrons detector): 15.0 kV, 8.7 mm; magn.: 500×, 1000×. The histograms of the mean size of powder grains with the fits of Gauss functions for GAp powder in the range of 10–100 μm (**C**) and below 10 μm (**D**).

The presence of CO_3_^2−^ and HPO_4_^2−^ groups in the specimens were verified by FTIR spectroscopy. In Figure A3a (Appendix A), the FTIR spectra for mineral apatites GAp and YAp were compared to the reference spectrum of commercially available HAp (nanoXIM HAp403). The reference spectrum gave a typical pattern for HAp [38] with a small content of carbonate groups, as was evidenced by asymmetric stretching vibration (ν_3_) and out-of-plane bending vibration (ν_2_) bands of CO_3_^2−^ in the spectral regions: 1400–1500 cm^−1^ for ν_3_ and around 875 cm^−1^ for ν_2_ [39,40]. The OH stretching band (at 3570 cm^−1^) and PO_4_ ν_3_ (~1090 cm^−1^ and ~1040 cm^−1^), ν_1_ (962 cm^−1^), ν_4_ (602 cm^−1^ and ~570 cm^−1^) and ν_2_ (472 cm^−1^) vibration bands were present in all samples, but the peak position of OH libration band (at 632 cm^−1^) was determined unambiguously only for nanoXIM HAp403. The fitting of carbonate group ν_3_ region (1600–1350 cm^−1^) with Gaussian distributions (Figure A3b, Appendix A), the absence of significant absorption intensity beyond 1500 cm^−1^ and the presence of ν_2_ band at 875 cm^−1^ indicated B type carbonate substitutions (CO_3_^2−^ for the PO_4_^3−^ groups) in the case of nanoXIM HAp403. However, the ν_3_ region in HAp spectrum (Figure A3b, Appendix A) showed some similarity to the spectra of Na-bearing carbonate apatite LM005 sample in [40] and to that of human enamel [40]. In GAp spectrum, the peak positions of carbonate group bands: ν_3_ (1449 cm^−1^, 1422 cm^−1^, see Figure A3c, Appendix A) and ν_2_ (858 cm^−1^) were also observed, suggesting the presence of B type substitutions. There is a little evidence of the presence of CO_3_^2−^ in YAp (compare spectra in Figure A3a, Appendix A). The trace amounts of CO_3_^2−^ and lack of HPO_4_^2−^ could not explain the low content of calcium cations in both mineral apatites; however, it should be pointed out that EDXS measurements might be subjected to relatively high errors (up to 10%). 

#### 2.1.2. Structural Characteristics of Mineral Apatites

**Figure A4:**
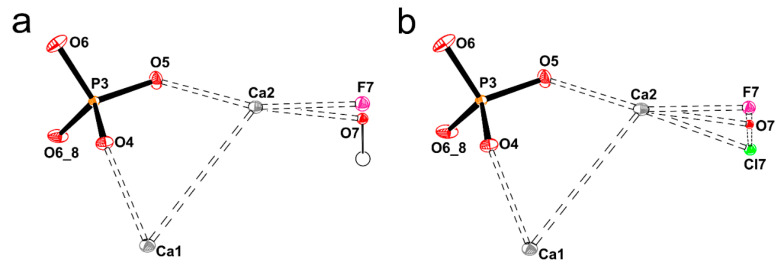
The drawing of the asymmetric unit contents of the apatites GAp (**a**) and YAp (**b**) with additional oxygen atom O6_8 to complete the phosphate group by the symmetry operation _8: x y z+½. The site occupancy factors of fluorine, chlorine atoms and hydroxyl groups are GAp 0.115(5) for F7, YAp 0.052(5) for O7, 0.091(2) for F7, 0.071(2) for O7 and 0.005(1) for Cl7.

**Figure A5:**
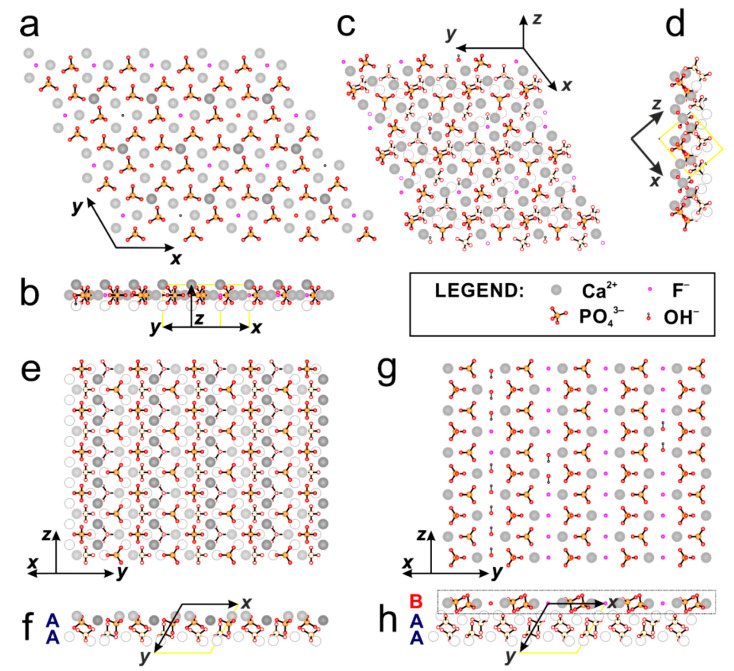
The structure of GAp shown as monolayers or double layers of atoms projected onto (001), (100) and (101) planes. (**a**) The double layer of atoms (of thickness Δz = ½*c*) projected onto (001). (**b**) The same layer viewed from the side along [210]. (**c**) The double layers of atoms projected onto (101). (**d**) The side view of (101) surface projected in [010] direction. Projection onto (100) plane in slab models of A-A type layers (PO_4_-rich surface) with Ca^2+^ and phosphate groups (**e**) and that of B type layer (Ca-rich surface) with F^−^ ions and OH^−^ groups (**g**). The side views of the layers showing sequence of A = Ca_3_(PO_4_)_2_ and B = Ca_4_(PO_4_)_2_(F,OH)_2_ for GAp (100) surfaces: ‘PO_4_-rich’ (**f**) and ‘Ca-rich’ (**h**), respectively. The layer projected on (**g**) is marked in black frame on (**h**). Color coding for atoms: calcium Ca1–dark grey, calcium Ca2–grey, oxygen–red, phosphorus–orange, fluorine–magenta and hydrogen–little black circle.

The structures of GAp and YAp were obtained from X-ray diffraction measurements for single-crystals. The asymmetric unit contents, as found from the refinement of the crystal structures of both apatites, were presented in Figure A4 (Appendix A). The hydroxyl group of GAp was partially substituted by F^−^ in the molar ratio OH/F = 0.62:1.38. In YAp, the substitution of hydroxyl group by both F^−^ and Cl^−^ anions was observed with the molar ratio OH/F/Cl = 0.86:1.09:0.05. The presence of Cl^−^ substitution in the YAp structure affected the unit cell parameters: lengthening *a* and shortening *c* periods, when compared with those for GAp. The refined chemical compositions were as follows: Ca_10_(PO_4_)_6_F_1.38_(OH)_0.62_ for GAp and Ca_10_(PO_4_)_6_F_1.09_(OH)_0.86_Cl_0.05_ for YAp. The details of experimental data refined atomic parameters (x, y, z, Uij) and site occupancy factors (s.o.f.) are summarized in Table A1, Table A2, Table A3, Table A4, Table A5 and Table A6 (Appendix A). The distribution of calcium cations, phosphate groups, fluoride anions and hydroxyl groups in the structure of apatite GAp is shown in Figure A5 (Appendix A) to emphasize the properties of the appropriate apatite plates used for cell cultures. 

The projections of crystal structure slabs reveal the arrangement of the most exposed atoms in {001}, {100} and {101} crystal faces corresponding to the prepared apatite plates. Figure A5a (Appendix A) shows the projection of the slab with the thickness of half of the unit cell (compare Figure A5b, Appendix A) onto (001), therefore do not reflect hexagonal symmetry of the apatites. However, in such a projection, three oxygen atoms, each of different PO_4_^3−^ group, and three Ca^2+^ cations are exposed around channel formed by F^−^ or OH^−^ anions following three-fold symmetry. The fragment of crystal structure projected onto (101) plane is illustrated in Figure A5c (Appendix A) (the thickness of the layer is presented in Figure A5d in respect to the unit cell) with the most exposed atoms colored. The PO_4_^3−^ groups, and thereby negative charges, are located on the set of parallelograms, while Ca^2+^ (positive charge) and F^−^ or OH^−^ (point negative charge) are located inside or in the center of the parallelogram. In case of the projection onto (100) plane, there are possibly at least two cross-sections shown in Figure A5e,g (Appendix A). According to [41], the apatite structure can be considered as build of layers parallel to *ac* showing the sequence –B–A-A–B– along [10], where A = Ca_3_(PO_4_)_2_ and B = Ca_4_(PO_4_)_2_(F,OH)_2_ (compare Figure A5f,h, Appendix A). In Figure A5e (Appendix A), the layers A-A (PO_4_-rich), shown in projection onto (100), are composed of parallel and alternating strips of Ca^2+^ and PO_4_^3−^ ions. Figure A5g (Appendix A) illustrates the layer B (Ca-rich) with the zigzag arrangement of Ca^2+^ and PO_4_^3−^ ions and anionic channels in between. The packing arrangement of those two (100) projections is different in comparison to the (001) and (101) ones. 

The proposed projections of GAp structure illustrate that only selected anions (F^−^ and OH^−^) from the ionic channels in the ratio corresponding to the chemical composition (F/OH = 1.38/0.62) are exposed on the surface. The projections of YAp structure are analogous to GAp, although the presence of Cl^−^ (F/OH/Cl = 1.09/0.86/0.05) may change local electrostatic distribution on the surface of slabs. 

The models of apatite structure of slabs proposed here are in agreement with the models proposed for HAp. In literature, usually the modelled surface {100} was also considered as “stoichiometric” slab, which is coplanar with (200) plane cross-section of A-A layers [11,41]. The surface energy calculated for HAp [11] was found to be the lowest for (001), whereas much higher values were obtained for “stoichiometric” (100) and for (101) surfaces (1.043, 1.709 and 1.646 J/m^2^, respectively). Additionally, electrostatic potential maps indicated higher activity of the (100) and (101). The experimental results of HRTEM imaging [42] showed that in vacuum the termination of {100} face of HAp was consistent with that cross-section, in which anionic channels are exposed (compare Figure A5g, Appendix A). On the other hand, the modelling of adsorption/desorption processes at {100} surface by HAp surface titration indicated that in both pure water and under physiological conditions, phosphate groups predominate at the surface, and the (200) plane (the “stoichiometric” one) was identified as the likely form of surface [43]. Main difference between our work and the earlier results found for HAp is that the properties of surfaces, such as hydrophobicity, surface energy and charge density, are modified by the fluoride and chloride ions present in the structure of mineral apatites and so influence the cellular response.

### 2.2. Cell–Mineral Apatite Interactions 

#### 2.2.1. Human Fibroblast Growth and Proliferation on Crystallographically-Oriented GAp and YAp Surfaces 

**Figure 1:**
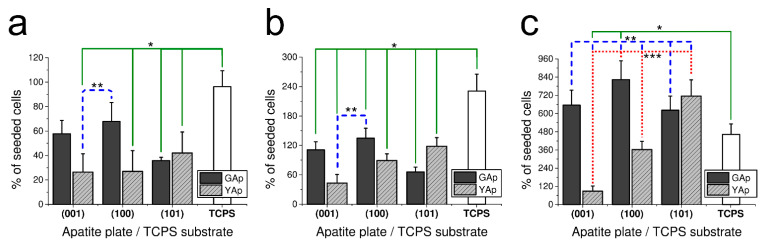
The fibroblast-cell growth-rate (calculated as a percentage of seeded cells) on apatite plates compared with TCPS substrate after 48 h (**a**), 3 days (**b**) and 5 days (**c**) of incubation. The number of cells seeded was 500 cells/mm^2^ in the case of (**a**) and 200 cells/mm^2^ in the case of (**b**,**c**). * Significant difference (*p* < 0.05) compared with TCPS control (continuous line). ** Significant difference (*p* < 0.05) between (001) YAp and remaining apatite plates (dashed line). *** Significant difference (*p* < 0.05) between (100) YAp and three other apatite plates: (001), (101) YAp and (100) GAp (dotted line). The bar graphs represent the mean ± SD of three independent experiments.

The results of fibroblasts incubation on the apatite plates of different crystallographic orientation after 2, 3 and 5 days are presented in Figure 1a–c, respectively. The number of cells grown on each apatite plate and on TCPS control are expressed as the percentage of the fibroblast number initially seeded onto substrates.

After 48 h of incubation on all tested apatite plates, a decrease in cell number compared with the number of initially seeded fibroblasts was observed. As presented in Figure 1a, significant differences were found between following surfaces: between TCPS and (001) and (101) GAp; between TPCS and (001), (100) and (101) YAp (all at *p* < 0.05); and between (100) GAp and (001) and (100) YAp (all at *p* < 0.05). The proliferation of fibroblasts grown on GAp apatite was more intense than on YAp (especially on (100) GAp surface), whereas on YAp, fibroblasts proliferated preferentially on (101) surface. The lowest cell amount among investigated GAp plates was counted on (101) and among YAp plates on (001) and (100). 

In the following experiment, the proliferation potency of fibroblasts grown on differently oriented plates of apatite GAp and YAp was evaluated after 3 days of incubation. The results presented in Figure 1b show the same tendency of cell growth as it was observed after 48 h of incubation. Similarly, the preferable surface of fibroblasts proliferation for GAp was (100) and for YAp (101) (however, the differences were not significant at *p* < 0.05). The minor cell growth was observed on (101) for GAp and (001) for YAp. The increase in number of cells compared with the initial cell amount was observed for all tested surfaces, but the proliferation rate on the apatite plates was lower than that measured on TCPS (with significant differences at *p* < 0.05 between each apatite sample and TCPS). 

In order to check the tendency of the fibroblast growth in longer period, the proliferation ratio was also measured after five days (Figure 1c). The most preferable substrates for cell growth were plates (100) GAp (no statistical differences at *p* < 0.05 compared with control) and (101) YAp (significant differences at *p* < 0.05 compared with control). The tendency of proliferation for GAp and YAp was preserved after 5 days, yet the number of cells on all tested apatite plates was higher than on TCPS. The percentage of living cells (in relation to the sum of living and damaged cells) was assessed by a Trypan Blue Exclusion Test at the end of each experiment. The viability of fibroblasts grown on apatite surfaces was as follows: 96 ± 4% for (001) GAp, 95 ± 5% for (100) GAp, 96 ± 5% for (101) GAp, 95 ± 4% for (001) YAp, 95 ± 5 % for (100) YAp, 93 ± 7% for (101) YAp and 96 ± 5% for TCPS (with no significant differences between all groups at *p* < 0.05).

#### 2.2.2. Cytotoxicity of GAp and YAp Surfaces 

**Figure 2:**
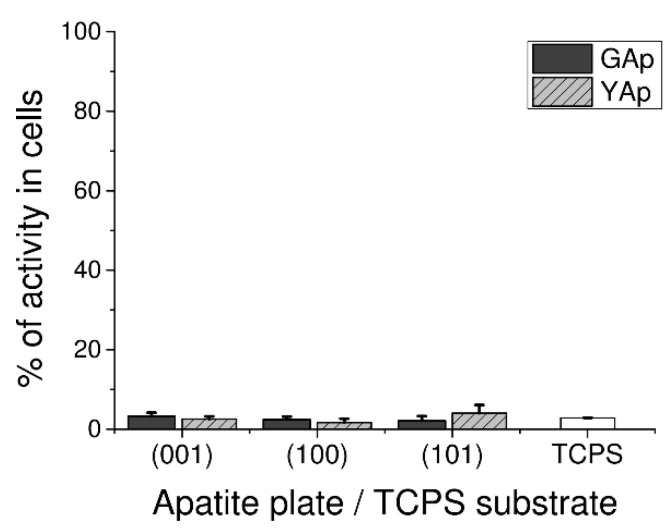
Cytotoxicity in human fibroblasts cultured on GAp and YAp apatite surfaces compared with TCPS substratum (control) and calculated after 48 h of incubation as the percentage of seeded cells. The cytotoxic scale is as follows: zero corresponds to non-toxicity, and 100% means the total leakage of LDH from cells (maximal toxicity). The density of seeded cells was 1 × 10^5^ cells/mL in 200 µL of medium. No significant differences exist between groups at *p* < 0.05 compared with each other and TCPS control.

LDH Assay used to assess GAp and YAp cytotoxicity revealed that none of tested surfaces exerted disrupting effect on cell membrane during 48 h of culture. Very low release of intracellular LDH from fibroblasts grown on apatite plates was measured: there were no significant differences between GAp and YAp plates at *p* < 0.05 and between apatite plates and TPCS at *p* < 0.05 (Figure 2). 

#### 2.2.3. Apoptosis and Necrosis of Cells Grown on GAp and YAp Surfaces 

**Figure 3:**
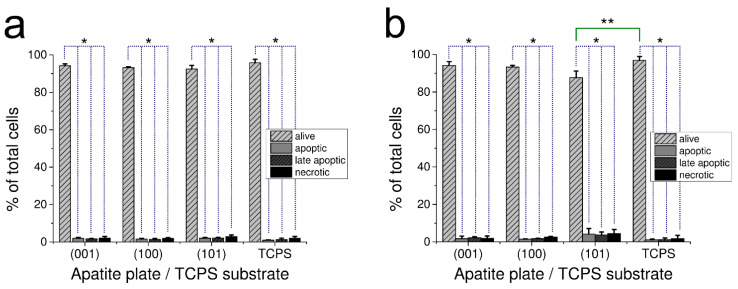
Effect of GAp (**a**) and YAp (**b**) apatite plates after 48 h of incubation on fibroblast apoptosis and necrosis. Control cells were grown on TPCS. The number of alive, apoptotic, late apoptotic or necrotic cells are expressed as the percentage of total cells that were grown on the plate of particular crystallographic orientation. The values are expressed as a mean percentage of three independent experiments with standard deviation. * Significant difference (*p* < 0.05) between alive and dead cells (dotted line). ** Significant difference (*p* < 0.05) between the alive cells of (101) YAp and TCPS (continuous line).

The effect of apatite plates on apoptosis and necrosis occurrence in the culture of human fibroblasts after 48 h is demonstrated in Figure 3a for GAp and in Figure 3b for YAp. The results of apoptotic, late apoptotic and necrotic cells are given as the percentage of total counted cells for each of the investigated samples. 

For all tested surfaces, the percentage of alive cells in population was very high (with no differences between GAp and YAp sets of plates at *p* < 0.05 and between the apatite plates and TPCS at *p* < 0.05). Only cells grown on (101) YAp had slightly lower percentage of alive cells in population than TPCS (significantly different at *p* < 0.05). The number of apoptotic/necrotic cells grown on this surface was low and comparable to the corresponding samples (with no differences between GAp and YAp plates at *p* < 0.05 as well as between the sets of apatite plates and TPCS at *p* < 0.05) (Figure 3). 

#### 2.2.4. Changes in Morphology of Fibroblasts Interacting with GAp and YAp Surfaces 

**Figure 4:**
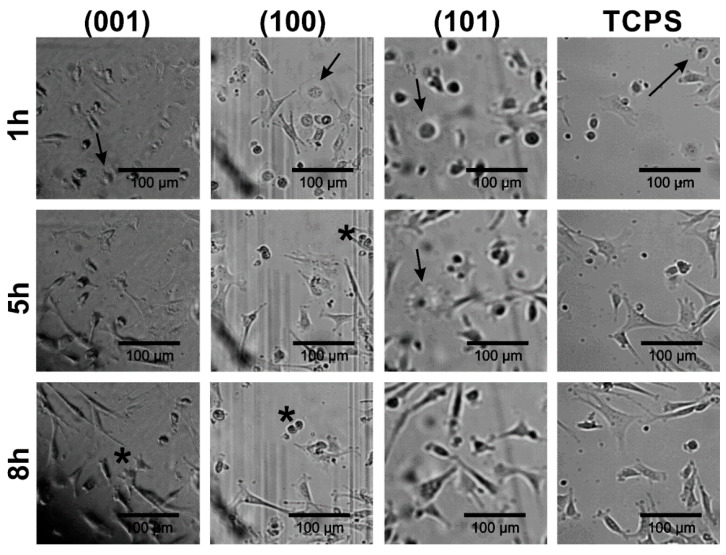
The fibroblast attachment to GAp plates of different crystallographic orientations compared with the reference substrate (TCPS), after 1 h, 5 h and 8 h of incubation. The cells at various stages of cytokinesis are marked with the asterisks. The cells in the beginning stage of cellular attachment to the surface are indicated with the arrows.

In order to assess if there are any differences in the fibroblast attachment during cell culture settlement between (001), (100) and (101) apatite plates as well as TCPS substrates, the morphology of cells cultured on GAp plates was documented using time-laps inverted microscopy (see Figure 4). It was observed that the process of cell–surface interactions on all tested apatite plates was similar to that on TCPS. However, the common feature of fibroblasts performing on all apatite plates was enhanced migration of cells compared to the migration of cells interacting with TCPS. One hour after cell seeding, fibroblasts were either polarized or stretched/spindle-shaped, and their morphology was typical for the early stage of the settlement. Additionally, the various stages of mitosis were observed (the representative cells during cytokinesis were marked with asterisks on Figure 4), both after 5 h and 8 h of incubation, indicating intensive cell division on apatite plates similar to that on TCPS. 

**Figure 5:**
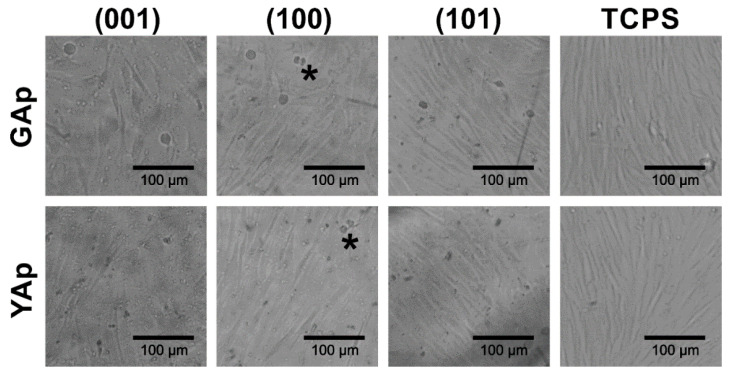
The fibroblasts morphology on the GAp and YAp plates and TCPS substrates after 48 h of incubation. The exemplary dividing cells are marked with the asterisks.

The microscopic observations after 48 h of incubation revealed variability in the morphology of the fibroblasts and in the cell density on each type of the apatite plate (Figure 5). On GAp (001) plate, the fibroblasts had a tendency to be stretched and arranged in a disordered way. By contrast, on GAp (100) and (101) plates (Figure 5, second and third photographs in the first row, respectively) the shape of the cells was regular and elongated, which is typical for fibroblasts observed in cell culture on TCPS (Figure 5, last column). The cells were well spread in one direction and mutually aligned. The similar cell arrangement was observed on all tested YAp plates (see Figure 5, second row). 

The changes of the cell morphology of fibroblasts after 3 and 5 days were similar to those after 48 h. On the fifth day of incubation, the confluence of all cell cultures on each tested surface reached 100% and fibroblasts covered all available surface area of the substrates.

#### 2.2.5. The Strength of Fibroblast Attachment to GAp and YAp Surfaces 

The experiments performed after 5 days of incubation revealed that the fibroblasts were more firmly attached to both GAp and YAp plates than to TCPS surface, with significant differences at *p* < 0.05 between apatites and TCPS (compare results shown in Table 2). Differences in the time of cell detachment between GAp and YAp plates were not statistically significant at *p* < 0.05. 

## 3. Discussion

Several studies had been published so far concerning eukaryotic cell–hydroxyapatite substratum interactions, but still very little is known about the effect of specific crystal surfaces on human cells. It should be emphasized here that many recent ongoing experiments involve tumor cells, yet the use of the cancer-cell models might result in the alteration of both metabolism and the function of cells. The performance of those cells does not resemble their normal counterparts in tissues and is considerably modified compared with the regular in vivo conditions. In our experiments, an original research model was adapted to examine the influence of single-crystal surfaces with defined crystallographic orientation on viability, growth and proliferation of fibroblasts, the most common cells derived from connective tissue in human body. Fibroblasts in cell culture exhibit shape polarity, so it could be consecutively observed how the elongated cells were dovetailed to the particular surface. 

In this work, we intended to show resultant cellular behavior on the substrates with different arrangement of ions exposed on the surfaces with various crystallographic orientation and not to study the influence of adsorbed protein layer on the living cells. However, there is a probable effect of competitive adsorption between proteins present in growth medium (originated from serum, such as bovine serum albumin, BSA and plasma fibronectin, pFN) and those synthesized by fibroblasts during incubation (such as cellular fibronectin, FN and collagens such as type I collagen, COLI) as intermediating factors in the process of cellular adhesion. The cell membrane protein adsorption depends on the electrostatic potential distribution of apatite surfaces [38,44]; thus, the principal factors are the chemical composition of single crystals and the arrangement of ions exposed on a particular apatite surface. Our evaluation of the number of ions per unit cell exposed on (100), (001) and (101) crystal faces of GAp and YAp apatites; the resultant charge of each surface; and the number of channel anions (containing OH^−^, F^−^ and/or Cl^−^) per 1000 nm^2^ were estimated on the basis of the most outer layers marked on GAp structure projections (see Figure A5, Appendix A). The results of estimation are given in Table 3.

According to the data presented above, the most positively charged surfaces are ‘PO_4_-rich’ (100) A and (101) ones, and the most negatively charged surface is the ‘Ca-rich’ (100) B surface. It should be noted that the protein as a whole responds to the differences of surface potential, but major interactions take place between particular protein groups (such as carboxylate groups) and ions (mostly Ca^2+^ in the case of apatites). In general, it was shown that inorganic surfaces were highly adhesive substrates for proteins [45].

It was observed for GAp surfaces that human fibroblasts settled down and attached to the oriented plates in analogous way as to TCPS substrate (Figure 4). After 48 h of growth, the cells on (001) GAp turned into stretched ones and lost their spindle shape (but without any change in morphology, which might have suggested transformation). Fibroblasts grown on the other tested surfaces and TCPS remained regular and elongated (Figure 5). The presence of additional ions in the apatite structure and the differences in their exposition on particular crystal faces might combine into the observed effect. In our study, the variations of surface potential distribution of substrates (compare Table 3) imply differences in fibroblasts performance on the apatite plates of different crystallographic orientations. 

The viability, growth and proliferation experiments aimed to determine if there are differences in the performance of fibroblasts seeded on the apatite substrates with respect to the crystallographic orientation of the plates. An interesting trend in the proliferation was observed: the cells on apatites grew slower at the beginning of experiment than on TCPS (the growth after 48 h and after 3 days was minor in all samples compared with TCPS, Figure 1a,b). The differences between cell proliferation on GAp and YAp plates and slower proliferation on the apatites compared with TCPS substrate after 48 h could be due to competitive adsorption between BSA and pFN on the surfaces. Recently, the influence of such interplay between BSA and pFN on fibroblast response was shown by Zelzer et al. [46]. After five days, fibroblasts on all tested GAp surfaces as well as on (101) YAp proliferated to higher extents than on the reference substrate (Figure 1c). Moreover, after 5 days of culture, when TCPS cell population had barely been doubled, fibroblasts cultured on the apatite plates reached higher proliferation ratio and still remained in the logarithmic stage of growth. The limited proliferation on TCPS after 5 days was visible, due to the contact inhibition of fibroblasts, while in parallel cultures on the (001), (100) and (101) surfaces of GAp and on the (101) of YAp, the cells still grew without disturbances in normal fibroblast appearance. The surface properties of particular crystal faces might have induced some changes in cell attachment, and consequently the apatite plates were capable of holding more fibroblasts than TCPS on the same growing area. The effect was accompanied by stronger cell attachment to the apatite plates than to TCPS, i.e., twice much time was required to detach cells adhered to the apatite than to the polymer substrates (compare Table 2). 

Concerning the relationship between cell proliferation and crystallographic orientation of the apatite plates, three observed tendencies should be emphasized: (1) cell proliferation is similar on (101) for both GAp and YAp, and it is correlated with high density of Ca^2+^ at the surface (see Table 3); (2) cell proliferation on (001) YAp is very small when compared to (001) GAp, and it is probably caused by Cl^−^ disturbance of surface potential distribution unfavorable for fibroblast performance; (3) cell proliferation on (100) GAp is twice that on (100) YAp, interpretation of such behavior is complex due to two possible arrangements of atoms at the surface (A or B type), and the decrease of cell number at (100) YAp might be a consequence of disturbance of surface potential distribution by Cl^−^ and/or different (100) cleavages. 

It is worth noting that the differences in protein adsorption could also be considered as dependent on the resultant charge of apatite surfaces and on the ratio of channel ions exposed on (001), (101) and (100) B surfaces of the apatites. However, the role of channel ions is negligible in the case of (100) A surfaces. According to Harding et al. [43], the surface (100) in buffered solution of growth medium is probably rich in phosphate groups and calcium cations (here, ‘PO_4_-rich’ A layer, Figure A5e,f, Appendix A); when exposed to the atmosphere (and in vacuum), it is terminated by B layer (compare Figure A5g,h, Appendix A). 

In present work, very low cytotoxicity of all tested apatite plates was demonstrated using three independent methods, including microscopic Trypan Blue Exclusion Test (see Section 2.2.1), colorimetric LDH release assay (Figure 2) and fluorescence apoptosis/necrosis quantitation (Figure 3). The molecular mechanism of harmful influence on cell survival may include apoptosis (type I cell death) and necrosis (type III cell death) phenomena. The cellular breakdown due to apoptosis as well as necrosis caused by biomaterial implemented into human body may exert detrimental effects, including enhanced loss of normally functioning cells (due to apoptosis) and excessive immune response (caused by necrosis). In our study, the overall tests confirmed that apatites were not harmful to cultured human normal fibroblasts. Only Annexin-V/7AAD assay, more sensitive than the others applied, revealed the slight decrease in the percentage of alive, biochemically active cells on (101) YAp surface. The effect was accompanied by very low percentage of apoptotic and necrotic cells, and the results suggest that none of molecular mechanisms, neither apoptosis nor necrosis, was particularly involved in decreasing the number of alive cells on (101) YAp (Figure 3). Liu et al. [47] reported enhanced apoptosis in human cell line grown on apatite, but those results were obtained using tumor (hepatic) cell line. Although the cytotoxic and proapoptotic effects of fluorides were reported [48], in our study the contents of F^−^ in (101) Yap were comparable to other tested surfaces (both Gap and Yap); therefore, the mild cytotoxicity of that surface was rather attributed to the presence of Cl^−^ (present in Yap and absent in Gap). Evidently, the decreased cell viability on (101) Yap was transient because after 5 days of incubation this surface was the second most preferred by fibroblasts (Figure 1c). Further discussion about potential influence of substitutions (and/or inclusions) present in the mineral samples on the cell response will be published elsewhere. 

Taken together, the phenomenon of fibroblast interaction with apatite crystallographically-oriented substratum is complex and requires further chemical and biological investigations. Regarding the model designed for the study, since no official recommendations and tests have been preferred, our assessments of apatite–cell interactions refer to state-of-art in the present investigations. In this context, the results of our studies might be useful in screening the effects of apatite crystallographically-oriented surfaces and in the evaluation detrimental/beneficial influence of apatite on living systems. Although similar investigations were conducted for biomaterials such as titanium [49,50]; texturized hydroxyapatite ceramics [14]; lithium-doped hydroxyapatites [51]; fluorapatite/glucan composites [52]; and rutile surfaces [53] using osteoblasts, preosteoblasts, and hepatocytes, to the best of our knowledge, the relation between the specific atomic structure of the surface and the normal human fibroblast biological performance is presented for the first time in our study. Despite recent advances in knowledge, few articles concern complex issues of potential HAp application in tissue engineering [54]. The presented interactions between human cells and apatite-crystal surfaces revealed the aspects of molecular mechanisms that may improve the process of evaluation of apatite bioavailability and advance the designing of biomaterials more suitable for medical interventions in tissue regeneration and controlled drug release [55].

## 4. Materials and Methods

### 4.1. Materials Used for Cell Culture 

Cell culture sterile equipment (plates, flasks, tips, centrifuge tubes, and pipettes) was from Sarstedt and from BD Biosciences. PBS without Ca^2+^ and Mg^2+^, pH 7.4 and non-enzymatic cell detach system were from PAA. Eagle’s minimum essential medium (EMEM) and fetal bovine serum (FBS) were from ATCC (Manassas, VA, USA). Penicillin, streptomycin and amphotericin mixture; Trypsin–0.05% EDTA solution; and Trypan Blue solution were from Sigma-Aldrich (Hamburg, Germany). Versenate (EDTA) was from Invitrogen. LDH test was from Biolabo (France). Flow cytometry chemicals were from BD Biosciences (Franklin Lakes, NJ, USA).

### 4.2. Preparation and Characteristics of Mineral Apatite Substrates 

The oriented plates cut from two types of apatite minerals were used in cell culture experiments. The geological apatites with hexagonal prismatic crystal habit originated from Slyudyanka, Lake Baikal, Russia (GAp) and from Imilchil, The Atlas Mountains, Morocco (YAp). The selected specimens of the crystals had well developed faces: {100} and {101} in the case of GAp, and {100}, {101} and {001} in the case of YAp. All apatite plates used in the experiments were cut parallel to natural crystal faces; ground to a thickness of about 1.0 mm; and finely polished using diamond particles (Struers) with diameters of 3 μm, 1 μm and 0.25 μm, consecutively. The plates cut from each mineral apatite single-crystal corresponded to (100), (001) and (101) crystallographic faces, i.e., they were parallel, perpendicular and oblique to 6-fold axis, respectively. 

Inclusions, imperfections and possible thickness heterogeneity of the apatite plates were documented using polarizing microscope (Micro SXT 0400003). Surface microstructure and chemical composition were determined using variable pressure (H_2_O vapour) scanning electron microscope (FEI E-SEM XL30) with energy dispersive X-ray spectrometer (EDAX GEMINI 4000) in the case of GAp (cut specimens), and SEM (HITACHI S-4700) with EDXS (NORAN VANTAGE) in the case of YAp (plates cut, polished and sputter coated with carbon using high vacuum Cressington Carbon Coater, 208 carbon). SEM Hitachi S-4700 with EDXS Noran Vantage-Si(Li) detector with 134 eV resolution and 30 mm^2^ active area enables the detection of elements down to boron. FEI E-SEM XL30 with EDAX Gemini 4000-Si(Li) detector with 133 eV resolution enables the detection of elements down to boron.

The FTIR spectra were recorded using the KBr pellet method for powdered (ground in ball mill) mineral apatites Gap and Yap, and for commercially available HAp powder-nanoXIM HAp403 (nano-sized hydroxyapatite spray-dryer 10.0 µm powder, Fluidi-Nova S.A.). The spectra were investigated using Equinox 55, Bruker in the range of 4000–400 cm^−1^ at a resolution of 2 cm^−1^. The phase purity of the powdered apatites was controlled by X-ray diffraction method (X’PertPRO, PANalytical diffractometer, Netherlands; CuKα: 1.5406 Å, 2θ-range: 10–70°, 0.02° resolution).

X-ray diffraction measurements for GAp and YAp were conducted at 20 °C on Nonius Kappa CCD single-crystal diffractometer, using monochromatic MoKα radiation (λ = 0.71073 Å). For intensity data collection, the fragments of the apatites, sized 0.30 mm × 0.19 mm × 0.15 mm (GAp) and 0.45 mm × 0.30 mm × 0.18 mm (YAp), were chosen. DENZO and SCALEPACK programs were used for intensity integrations, lattice parameter refinement and data reduction (Lorentz, polarization and absorption corrections, and scale factors). The space groups in both cases were determined as *P* 6_3_/m (hexagonal). The crystal lattice parameters were as follows: *a* = 9.3839(4), *c* = 6.8867(3) Å for GAp; and *a* = 9.4058(4), *c* = 6.8807(3) Å for YAp. Crystal structure solutions were done using SIR92 program [56]. The refinement of structural parameters, 42 for GAp and 44 for YAp, performed on the basis of 548 and 432 F^2^(hkl) data, respectively, using SHELXL97 [57], was converged to the final R-factors: R1 = 0.0191, *w*R2 = 0.0492 and S = 1.094 for GAp and R1 = 0.0208, *w*R2 = 0.0525 and S = 1.121 for YAp. The crystal structure drawings were prepared using ORTEP-3 for Windows [58].

### 4.3. Cell Culture Experiments

#### 4.3.1. Preparation of the Substrata for Cell Culture

The transparent plates were prepared from the apatites to shape the cell-culture well. Prior to the consecutive cell culture experiments, the apatite plates were polished, washed thoroughly in ultrapure water (Milli-Q, Millipore, Burlington, MA, USA) and steam-sterilized (autoclave ASV 400 × 600 Vertical Steam Sterilizer, Poland) for 20 min. at 121 °C under pressure of 1.52 bar (1.5 atm). After the sterilization process, the plates were checked for optical transparency under polarizing microscope. Then, plates were inserted at the bottom of multi-well tissue culture plates.

#### 4.3.2. Cell Culture Conditions

Human fibroblasts were obtained from American Type Cell Culture collection, ATCC (ATCC designation: BJ, CRL-2522, normal adherent cells from male, *Homo sapiens*). Cells purposed for experiments were between 10th and 20th passages. Fibroblasts were thawed and then suspended in complete growth medium EMEM supplemented with antibiotic solution containing 100 IU/mL penicillin, 0.1 mg/mL streptomycin, 0.25 μg/mL amphotericin (Sigma-Aldrich, Hamburg, Germany) and 10% *v*/*v* FBS. Cells were maintained as monolayers at 37 °C in humidified atmosphere of 5% CO_2_ in air and were subcultured using Trypsin-0.05% EDTA solution twice a week. The percentage of living cells was controlled during the culture by a Trypan Blue Exclusion test (0.4% Trypan Blue solution in buffered PBS, pH 7.4). The cell morphology was investigated by inverted light microscope (Olympus CKX 41SF-5 microscope and CAM-UV 30 camera, Olympus, Hamburg, Germany). The total number of fibroblasts were counted in automatic cell counter (Countess, Invitrogen, Waltham, MA, USA). The amount of suspension for each experiment was assigned in preliminary tests provided the homogenous distribution of the settling cells over the plates. The number of cells attached to TCPS substrates and apatite plates after 24 h was evaluated and on average reached 97–100% of seeded cells.

Initially, plates sintered from commercially available HAp (nanoXIM HAp403), obtained by the method described in [59], had been evaluated as the reference substrate for the fibroblast cell culture. However, it was impossible to precisely assess the area of cell growth since the plates did not appear to be sufficiently transparent. Therefore, their use as control for the planned experiments was not adequate. Thus, the growth of fibroblasts on the tissue culture grade polystyrene (TCPS) surface, commonly used for cell culturing, had been chosen as the appropriate reference positive control (compare to [60]). Each experiment was repeated three times.

#### 4.3.3. Cell Proliferation/Viability Experiments 

Cells were suspended in the complete growth medium and seeded onto the apatite plates of different crystallographic orientations as well as on the reference TCPS plate. In the first experiment, the fibroblast incubation with apatite plates was continued for two days, in the second one-for three days, and in the third one-for five days. For 48 h incubation, the initial cell density was 500 cells/mm^2,^ and for 3- and 5-days incubation, 200 cells/mm^2^. During the third experiment, the medium was changed after three days into a new EMEM with amount of FBS decreased to 1%. At the end of every experiment, media and cells from each plate were collected and centrifuged at 250× *g* for 5 min., suspended in the defined volume of buffered PBS (pH 7.4) and counted in automatic cell counter (Countess, Invitrogen, Waltham, MA, USA). Trypsin-0.05% EDTA solution was used to detach fibroblasts from all surfaces. Following each incubation, the viability of fibroblasts in each sample was assessed by a Trypan Blue Exclusion Test. The cells that did not accept blue dye were considered as viable and counted. The results were given as the percentage of viable cells of total counted cells.

#### 4.3.4. Cytotoxicity Assay (Lactate Dehydrogenase Test, LDH Test)

The cytotoxicity of apatites measured as LDH leakage was determined by using commercially available kit (Biolabo, Maizy, France). For LDH test, fibroblasts were seeded into 96-well plate at a density of 1 × 10^5^ cells/mL in 200 µL of medium onto the apatite plates of different crystallographic orientation as well as on the reference TCPS plates (control). Following 48 h of incubation, medium was collected and cells were lysed (Triton-X, 1% *v*/*v*, Sigma-Aldrich, Germany) to release the intracellular LDH. Then, NADH concentration generated in the reaction of the reduction of pyruvate was measured at 340 nm using BMG Labtech’s FLUOstar Optima microplate reader (Germany) according to manufacturer protocol. For each sample, the result was given as the percentage of LDH in the medium versus total LDH activity in the cells. 

#### 4.3.5. Cell Apoptosis and Necrosis Quantitation (Annexin-V/7AAD Assay)

Cells were suspended in the complete growth medium (1 × 10^5^/mL in 200 µL of medium) and seeded onto the apatite plates of different crystallographic orientation as well as on the reference TCPS plate. After 48 h of incubation, fibroblasts were collected and analyzed on a LSRII flow cytometer, using FACSDiva software (BD Biosciences Immunocytometry Systems, USA). Fluorescent dyes 7-Aminoactinomycin D (7-AAD, excitation/emission 543/647 nm) and Annexin-V (excitation/emission 490/515 nm) were used. The cells were gated according to forward (FSC), side scatter (SSC) and appropriate fluorescence parameters. The alive cells were negative for Annexin-V and 7-AAD, the apoptotic cells were defined as Annexin-V-positive and 7-AAD-negative, the late apoptotic cells were Annexin-V- and 7-AAD-positive, and the necrotic cells were Annexin-V-negative and 7-AAD-positive [61]. The results were given as the percentage of alive, apoptotic, or necrotic cells of total counted cells.

#### 4.3.6. The Morphology of Cells Attached and Grown on Apatite Plates

The cell attachment during first 8 h of incubation was documented using time-laps microscope JuLi Smart Fluorescence Cell Imager (Digital Bio Technologies, Cambridge, MA, USA). The fibroblast morphology and mutual cell arrangement on the apatite plates during experiments were inspected and registered using inverted light microscope (CK40F Olympus) and compared with the control substrates (TCPS). Cells were not fixed or stained, to not disturb their expected subtle morphology and mutual arrangement changes.

#### 4.3.7. The Strength of Cell Attachment to the Specific Apatite Surfaces

Fibroblasts were seeded onto the plates (200 cells/mm^2^) and incubated for five days as described in Section 4.3.3. The strength of attachment of fibroblasts to the apatite plates was evaluated in comparison to that observed for the TCPS substrates, using three independent tests: enzymatic system (Trypsin-0.05% EDTA solution), non-enzymatic systems and versenate. For each apatite plate and for TCPS substratum, the specific time interval needed for the detachment of cells was measured. 

### 4.4. Statistical Evaluation 

All experiments were conducted at least in triplicate and expressed as the mean ± standard deviation (SD). Statistical significance analysis was performed using Origin 9.0 software by one-way analysis of variance (ANOVA) followed by the Tukey post-hoc multiple comparison test. The levels of *p* < 0.05 were considered as statistically significant.

## 5. Conclusions

In the present work, the bulk and the surfaces of the GAp and YAp plates were characterized at a microscopic level by the SEM-EDXS technique and at the atomic level by the single-crystal X-ray diffraction method. Both mineral apatites used in our experiments were fluorhydroxyapatite with calcium cations deficiency (Ca/P ratio < 1.67) and with the trace amount of carbonate groups, which was confirmed by FTIR spectroscopy. Additionally, YAp had chloride anion substitution in the ionic channel, which distorted the crystal lattice in comparison to GAp. The surfaces of apatite plates were, in general, smooth at the microscopic level, as shown by SEM. The particular crystal faces of both apatites were characterized by the distribution of calcium cations, phosphate groups and fluoride/hydroxyl/chloride anion ratio. The surface with the predominating exposition of channel ions that was at the same time the most negatively charged was identified as (100) B. On the other hand, the most positively charged surfaces, with exposed calcium cations, were (101) and (100) A. The (001) surface was either neutral or slightly negatively charged, with one channel ion per unit cell content. The type of ions, particularly those from the ionic channel, exposed at the surface might have a significant impact on the local surface properties of (001) due to the considerable differences in spatial atomic environment of F^−^, OH^−^ and Cl^−^ anions. 

The main results of the studies point to (100) GAp as the surface having the most favorable effect on fibroblast proliferation, whereas (001) YAp has a disadvantageous impact on the proliferation pattern of cells after a longer incubation time, probably due to the influence of chloride ions, which are mainly exposed on the (001) YAp surface. 

## Figures and Tables

**Table 1:** Mean chemical composition of the apatites GAp and YAp as found by SEM-EDXS. HAp refers to the chemical composition of stoichiometric hydroxyapatite: Ca_10_(PO_4_)_6_(OH)_2_.

Chemical Element	HAp wt.%	GAp * wt%				YAp ** wt%			
		(001)	(100)	(101)	Mean	(001)	(100)	(101)	Mean
**Ca**	**39.9**	38.3	36.9	41.8	**39.0**	40.8	41.3	40.5	**40.9**
**P**	**18.5**	18.5	17.7	16.6	**17.6**	17.2	17.1	17.3	**17.2**
**F**		2.7	6.2	2.0	**3.6**	2.3	2.0	2.9	**2.4**
**Cl**		–	–	–	**–**	0.7	0.7	0.6	**0.7**
**Si**		0.7	0.6	0.9	**0.7**	0.5	0.3	0.2	**0.3**
**S**		0.6	0.9	0.6	**0.7**	–	–	–	**–**
**O**	**41.4**	39.2	37.7	38.1	**38.4**	38.5	38.6	38.5	**38.5**
**H**	**0.2**								

* For GAp, Na, Mg, Al (below 0.5 wt%) and carbon (<5 wt%) were found; ** for YAp, the presence of Na and S (below 0.3 wt%) were found; and the amount of C was not measured due to the method of carbon coating the apatite plates (see Section 4.2).

**Table 2:** The time of cell detachment in relation to the type of growth surface. The averaged time of cell detachment (min) from each of the substratum ± SD is given for enzymatic system and non-enzymatic system, and for versenate. The means with unlike superscript letters are significantly different (for ^a^ and ^b^ *p* < 0.05).

Type of Surface	Estimated Time of Cell Detachment (min)		
	Enzymatic System	Non-Enzymatic System	Versenate
**TCPS**	5 ± 2 ^a^	3 ± 1 ^a^	10 ± 5 ^a^
**GAp**	12 ± 3 ^b^	7 ± 2 ^b^	24 ± 5 ^b^
**YAp**	12 ± 3 ^b^	8 ± 3 ^b^	23 ± 5 ^b^

**Table 3:** Evaluation of ions/groups number per unit cell on the outermost layer for the following apatite crystal faces: {100}, {001} and {101}, and the approximate number of hydroxyls, fluoride, and chloride anions per surface area of 1000 nm^2^ for GAp and YAp. Faces (100) A and (100) B corresponds to the ‘PO_4_-rich’ (Ca/P = 1.5 < 1.67) and ‘Ca-rich’ (Ca/P = 2.0 > 1.67) cases, respectively (compare Figure A5 Appendix A).

Apatite Crystal Face	OH^−^/F^−^/Cl^−^	Ca^2+^	PO_4_^3−^	Resultant Charge	No of OH^−^/F^−^/Cl^−^/1000 nm^2^	
					GAp	YAp
**(001)**	**1**	**4**	**3**	2−	406/905/---	561/711/33
**(101)**	1.5	7	4	1.5+	580/1291/---	805/1020/46
**(100) A**	---	4	2	2+	---	---
**(100) B**	2	2	2	4−	959/2136/---	1329/1685/77

## Data Availability

Data is contained within the article.

## Appendix A

**FTIR spectra and powder diffraction data for mineral apatites**

The FTIR spectra of powdered mineral apatites GAp and YAp in the range of 4000–400 cm^−1^ were measured. The positions of vibration bands for PO_4_ and CO_3_ groups, as well as those of symmetric stretching vibration and libration bands for OH, were assigned. The absorbance of OH libration and vibration bands was not as pronounced for GAp and YAp as it was found for pure hydroxyapatite (HAp). This means that the mineral apatites have lower contents of hydroxyl groups compared to HAp.

The powder diffraction patterns for GAp and YAp were compared with the records present in PDF-2 database (International Center for Diffraction Data, Newtown Square, PA, USA) for hydroxyapatite, fluorapatite and chlorapatite. The data were selected for the mineral samples of different origins: Holly Springs in Georgia, USA, Durango in Mexico, and Kragero in Norway, with chemical compositions: Ca_10_(PO_4_)_6_(OH)_2_, Ca_10_(PO_4_)_6_F_1.88_Cl_0.20_ and Ca_10_(PO_4_)_6_F_0.18_Cl_1.76_, respectively. The positions and relative intensities of diffraction maxima calculated for samples correspond to hydroxy- and fluorapatites and are distinct from those for chlorapatites. Any differences in diffraction maxima intensities were assigned to the same texture of the powders. All mineral samples showed a high degree of crystallinity, as is indicated by the narrow widths of the diffraction maxima.

**Grain diameter of powdered apatites**

The mean diameters of grains for powdered GAp and YAp were estimated based on SEM micrographs (exemplary micrographs are shown below, Appendix A Figure A3Y(A,B)). The distribution of the mean grain diameters was found to be bimodal for all apatite powders. Typical histograms are shown in Appendix A Figure A3Y(C) in the range 10–100 μm and in Appendix A Figure A3Y(D) below 10 μm. The distributions were fitted with Gauss function in Origin Pro 9.0 programme (Northampton, MA, USA), and the mean diameters were found to be around 20 and 3 µm for all apatite powders.

**Chemical composition and structural data for GAp and YAp**

**Table A1:** Mean chemical composition of mineral apatites GAp and YAp evaluated from SEM-EDXS measurements. The data are compared to the chemical composition of biological apatites (enamel and bone apatites) and mineral apatites originated from the same locations as GAp and YAp.

Apatite Name	Apatite Origin	Chemical Composition			Presence of CO_3_^2−^	Ca/P Ratio
		Cations	Anions	Ionic Channels		
**Mineral apatites**						
**GAp**	Slyudyanka, Russia	Ca_9.41_Na_0.10_Mg_0.09_	(PO_4_)_5.55_(SiO_4_)_0.28_(SO_4_)_0.17_	F_1.38_(OH)_0.62_	traces ^a^	1.70
**YAp**	Imilchil, Morocco	Ca_9.86_	(PO_4_)_5.87_(SiO_4_)_0.13_	F_1.22_(OH)_0.62_Cl_0.16_	traces ^a^	1.68
**Biological Apatites**						
**E-Ap** **^b^**	enamel	Ca_9.52_Na_0.30_Mg_0.18_	(PO_4_)_5.85_(CO_3_)_0.30_	(OH)_1.34_(CO_3_)_0.33_Cl_0.07_□_0.26_	3–8 wt%	1.63
**B-Ap ^c^**	bone	Ca_8.3_□_1.7_	(PO_4_)_4.3_(HPO_4_ and CO_3_)_1.7_	(OH and/or CO_3_)_0.3_□_1.7_	---	<1.67
**Mineral Apatites**						
**S-Ap ^d^**	Slyudyanka, Russia	Ca_9.80_Na_0.08_Mg_0.09_K_0.01_	(PO_4_)_5.88_(SiO_4_)_0.01_(SO_4_)_0.01_ (CO_3_)_0.03_	F_1.25_(OH)_0.73_Cl_0.03_	<0.1 wt%	1.67
**HAM-Ap ^e^**	High Atlas, Morocco	Ca_9.90_Na_0.04_Sr_0.02_ RE_0.04_	(PO_4_)_5.80_(SiO_4_)_0.10_(SO_4_)_0.06_	F_1.34_(OH)_0.56_Cl_0.10_	none	1.71

**Notes:**^a^ Found by FTIR. ^b^ Proposed by Simmer and Fincham [62]. ^c^ As proposed by Cazalbou et al. [63]. ^d^ Calculated from chemical composition (wt.%) found by Chaikina and Aman [64]. ^e^ Calculated from chemical composition (wt.%) found by Henderson [65].

**Table A2:** Crystallographic data, diffraction measurement conditions and structure refinement parameters for mineral apatites GAp and YAp.

Ca_10_(PO_4_)_6_(OH,F,Cl)_2_	GAp Slyudyanka, Russia	YAp Imilchil, Morocco
Empirical formula	Ca_10_(PO_4_)_6_(F_1.38_OH_0.62_)	Ca_10_(PO_4_)_6_(Cl_0.05_F_1.09_OH_0.86_)
Formula weight Mr	1007.38	1007.73
Temperature T/K	293(2)	293(2)
Wavelength λ/Å	0.71073	0.71073
Crystal system	hexagonal	hexagonal
Space group *	*P* 6_3_/m, ITC No176	*P* 6_3_/m, ITC No176
Unit cell dimensions/Å,°	*a* = *b* = 9.3839(4) *c* = 6.8867(3) α = β = 90, γ = 120	*a* = *b* = 9.4058(4) *c* = 6.8807(3) α = β = 90, γ = 120
Unit cell volume V/Å^3^	525.18(4)	527.17(4)
Z; D_X_/Mg m^−3^	1; 3.185	1; 3.174
Absorption coefficient μ/mm^−1^	3.091	3.084
F(000)	500	500
Crystal size/mm	0.30 × 0.19 × 0.15	0.45 × 0.30 × 0.18
Absorption corrections	Semi-empirical from equivalents	
Max. and Min. transmission	0.6543, 0.4574	0.6067, 0.3375
Theta range θ/°	3.88 – 29.96	3.88 – 27.45
Index ranges	h: 0,14; k: −10,0; l: −9,5	h: 0,12; k: −10,0; l: −6,8
Reflections collected/symmetrically independent [R_int_]/observed [I > 2σ_(I)_]	794/548 [R_int_ = 0.0150] 520	616/433 [R_int_ = 0.0170] 422
Data completeness/%	99.3	98.9
Refinement method	Full-matrix least-squares on F^2^	
Data/restraints/parameters	548/0/42	433/1/47
S—Goodness-of-fit on F^2^	1.094	1.190
Final R indices [I>2σ_(I)_]	R1 = 0.0191 *w*R2 = 0.0484	R1 = 0.0224 *w*R2 = 0.0556
Final R indices [all data]	R1 = 0.0208 *w*R2 = 0.0492	R1 = 0.0230 *w*R2 = 0.0559
Extinction coefficient	0.028(3)	0.090(5)
Δρ_max_, Δρ_min_, rms/eÅ^−3^	0.364, −0.548, 0.083	0.394, −0.850, 0.123

**Note:** * ITC corresponds to International Tables for Crystallography space group description [66].

**Table A3:** Fractional atomic parameters x, y, z and equivalent displacement parameters Ueq (Å^2^) defined as 1/3 orthogonalized symmetrical displacement tensor Uij. The value of refined site occupancy factor is given in brackets following Wyckoff position (space group *P* 6_3_/m, ITC No 176 [66]).

**GAp**					
**ATOM**	**Positions**	**x**	**y**	**z**	**Ueq**
Ca(1)	4 *f* 3.	0.6667	0.3333	0.50114(7)	0.01022(15)
Ca(2)	6 *h* m.	0.99278(5)	0.24217(5)	0.7500	0.00854(14)
P(3)	6 *h* m.	1.02934(6)	0.63108(6)	0.7500	0.00581(15)
O(4)	6 *h* m.	0.84261(18)	0.51609(18)	0.7500	0.0106(3)
O(5)	6 *h* m.	1.12065(19)	0.53326(19)	0.7500	0.0122(3)
O(6)	12 *i* 1	1.08454(13)	0.74258(13)	0.92936(16)	0.0145(3)
F(7)	$2\;a\;\bar{6}$. (0.69)	1.0000	0.0000	0.7500	0.0104(10)
O(7)	4 *e* 3. (0.16)	1.0000	0.0000	0.692(2)	0.006(2)
H(7)	4 *e* 3. (0.16)	1.0000	0.0000	0.54(3)	0.007(3)
**YAp**					
**ATOM**	**Positions**	**x**	**y**	**z**	**Ueq**
Ca(1)	4 *f* 3.	−0.3333	0.3333	0.00132(9)	0.0099(2)
Ca(2)	6 *h* m.	−0.00678(6)	0.24406(6)	0.2500	0.0091(2)
P(3)	6 *h* m.	0.02978(8)	0.63108(8)	0.2500	0.0061(2)
O(4)	6 *h* m.	−0.1567(2)	0.5153(2)	0.2500	0.0110(4)
O(5)	6 *h* m.	0.1218(2)	0.5342(2)	0.2500	0.0132(4)
O(6)	12 *i* 1	0.08502(17)	0.74239(16)	0.4296(2)	0.0160(3)
F(7)	$2\;a\;\bar{6}$. (0.54)	0.0000	0.0000	0.2500	0.0086(18)
O(7)	4 *e* 3. (0.21)	0.0000	0.0000	0.183(3)	0.0033(19)
Cl(7)	4 *e* 3. (0.01)	0.0000	0.0000	0.083(12)	0.006(9)

**Table A4:** Anisotropic displacement parameters Uij (Å^2^). T = exp{−2π^2^ [ h^2^ *a**^2^ U11 + … + 2 h k *a* b** U12 ]}.

**GAp**						
	**U11**	**U22**	**U33**	**U23**	**U13**	**U12**
Ca(1)	0.0121(2)	0.0121(2)	0.0065(2)	0	0	0.0060(1)
Ca(2)	0.0083(2)	0.0096(2)	0.0076(2)	0	0	0.0043(2)
P(3)	0.0052(2)	0.0055(2)	0.0058(2)	0	0	0.0020(2)
O(4)	0.0067(6)	0.0108(7)	0.0103(7)	0	0	0.0015(6)
O(5)	0.0090(7)	0.0101(7)	0.0191(8)	0	0	0.0060(6)
O(6)	0.0129(5)	0.0125(5)	0.0101(5)	−0.0043(4)	0.0029(4)	0.0003(4)
**YAp**						
	**U11**	**U22**	**U33**	**U23**	**U13**	**U12**
Ca(1)	0.0117(3)	0.0117(3)	0.0063(4)	0	0	0.0059(1)
Ca(2)	0.0078(3)	0.0112(3)	0.0073(3)	0	0	0.0041(2)
P(3)	0.0055(3)	0.0063(3)	0.0058(4)	0	0	0.0023(2)
O(4)	0.0068(9)	0.0113(9)	0.0106(9)	0	0	0.0013(7)
O(5)	0.0103(9)	0.0117(9)	0.0201(10)	0	0	0.0073(8)
O(6)	0.0139(7)	0.0138(7)	0.0107(7)	−0.0053(5)	0.0031(5)	−0.0003(5)

**Table A5:** Comparison of selected bond length [Å] for GAp and YAp.

**BOND**	**GAp**	**YAp**	
Ca(1)-O(4)	2.4027(11)#1	2.4040(13)#1	
Ca(1)-O(4)	2.4027(11)	2.4040(13)	
Ca(1)-O(4)	2.4027(11)#2	2.4040(13)#2	
Ca(1)-O(5)	2.4583(11)#3	2.4541(14)#3	
Ca(1)-O(5)	2.4583(11)#4	2.4541(14)#4	
Ca(1)-O(5)	2.4583(11)#5	2.4541(14)#5	
Ca(1)-O(6)	2.8045(12)#6	2.8051(15)#6	
Ca(1)-O(6)	2.8045(12)#7	2.8051(15)#7	
Ca(1)-O(6)	2.8045(12)#8	2.8051(15)#8	
Ca(2)-F(7)	2.3071(4)	2.3281(5)	
Ca(2)-O(7)	2.342(3)	2.374(4)	
Ca(2)-O(7)	2.342(3) #9	2.374(4) #9	
Ca(2)-Cl(7)		2.60(4)	
Ca(2)-Cl(7)		2.60(4) #9	
Ca(2)-O(6)	2.3509(11)#6	2.3476(14)#10	
Ca(2)-O(6)	2.3509(11)#10	2.3476(14)#11	
Ca(2)-O(5)	2.3715(16)	2.3690(19)	
Ca(2)-O(6)	2.4989(11)#11	2.5031(13)#6	
Ca(2)-O(6)	2.4989(11)#12	2.5031(13)#12	
Ca(2)-O(4)	2.6882(17)#1	2.705(2) #1	
P(3)-O(6)	1.5319(11)	1.5329(14)	
P(3)-O(6)	1.5319(11)#9	1.5329(14)#9	
P(3)-O(4)	1.5310(15)	1.5337(19)	
P(3)-O(5)	1.5373(16)	1.5380(19)	
Symmetry conditions used to generate symmetrically related atoms:			
**GAp**	#1 −x + y+1, −x + 1, z	#2 −y + 1, x − y, z	#3 x − y, x − 1, −z + 1
	#4 −x + 2, −y + 1, z + 1	#5 y, −x + y + 1, −z + 1	#6 −x+2, −y + 1, z − 1/2
	#7 x − y, x − 1, z − 1/2	#8 y, −x + y + 1, z − 1/2	#9 x, y, −z + 3/2
	#10 −x + 2, −y + 1, −z + 2	#11 −y + 2, x − y, z	#12 −y + 2, x − y, −z + 3/2
**YAp**	#1 x − y − 1, x, z + 1/2	#2 y, −x + y + 1, z + 1/2	#3 −x + y, −x, −z − 1/2
	#4 x, y + 1, −z − 1/2	#5 −y − 1, x − y, −z − 1/2	#6 −y, x – y + 1, z − 1
	#7 −x, −y, −z − 1	#8 −x + y − 1, −x, z − 1	#9 y, −x + y, −z + 1
	#10 y + 1, −x + y, z + 1/2	#11 x – y + 1, x + 1, −z + 1	#12 x, y + 1, −z + 1/2

**Table A6:** Geometry of hydrogen bonds in GAp [Å, °].

**D-H…A**	**Position of A**	**D-H H…A D…A**	**DHA**
O(7)-H(7)…O7	−x + 2, −y, −z + 1	1.1(2) 1.6(2) 2.64(3)	180
O(7)-H(7)…F7	−x + 2, −y, −z + 1	1.1(2) 2.0(2) 3.04(2)	180
Symmetry conditions used to generate symmetrically related atoms:			
**GAp**	#1 −x + y + 1, −x + 1, z	#2 −y + 1, x − y, z	#3 x − y, x − 1, −z + 1
	#4 −x + 2, −y + 1, z + 1	#5 y, −x + y + 1, −z + 1	#6 −x + 2, −y + 1, z − 1/2
	#7 x − y, x − 1, z − 1/2	#8 y, −x + y + 1, z − 1/2	#9 x, y, −z + 3/2
	#10 −x + 2, −y + 1, −z + 2	#11 −y + 2, x − y, z	#12 −y + 2, x − y, −z + 3/2
**YAp**	#1 x – y − 1, x, z + 1/2	#2 y, −x + y + 1, z + 1/2	#3 −x + y, −x, −z − 1/2
	#4 x, y + 1, −z − 1/2	#5 −y − 1, x − y, −z − 1/2	#6 −y, x − y + 1, z − 1
	#7 −x, −y, −z − 1	#8 −x + y−1, −x, z−1	#9 y, −x + y, −z + 1
	#10 y + 1, −x + y, z + 1/2	#11 x − y + 1, x + 1, −z + 1	#12 x, y + 1, −z + 1/2

## References

[1] Cacciotti I. (2019). Multisubstituted Hydroxyapatite Powders and Coatings: The Influence of the Codoping on the Hydroxyapatite Performances. Int. J. Appl. Ceram. Technol..

[2] Haider A., Haider S., Han S.S., Kang I.K. (2017). Recent Advances in the Synthesis, Functionalization and Biomedical Applications of Hydroxyapatite: A Review. RSC Adv..

[3] Arcos D., Vallet-Regí M. (2020). Substituted Hydroxyapatite Coatings of Bone Implants. J. Mater. Chem. B.

[4] Li K., Chen J., Xue Y., Ding T., Zhu S., Mao M., Zhang L., Han Y. (2021). Polymer Brush Grafted Antimicrobial Peptide on Hydroxyapatite Nanorods for Highly Effective Antibacterial Performance. Chem. Eng. J..

[5] Chaudhary S., Sharma P., Renu, Kumar R. (2016). Hydroxyapatite Doped CeO_2_ Nanoparticles: Impact on Biocompatibility and Dye Adsorption Properties. RSC Adv..

[6] Turnbull G., Clarke J., Picard F., Riches P., Jia L., Han F., Li B., Shu W. (2018). 3D Bioactive Composite Scaffolds for Bone Tissue Engineering. Bioact. Mater..

[7] Wang W., Yeung K.W.K. (2017). Bone Grafts and Biomaterials Substitutes for Bone Defect Repair: A Review. Bioact. Mater..

[8] Dorozhkin S.V. (2009). Calcium Orthophosphates in Nature, Biology and Medicine. Materials.

[9] Xu Z., Xia Y., Zhou P., Li J.J., Yang M., Zhang Y., Zhang Y., Xie Y., Li L., Pan H. (2021). Silicon Incorporation into Hydroxyapatite Nanocarrier Counteracts the Side Effects of Vancomycin for Efficient Chronic Osteomyelitis Treatment. Chem. Eng. J..

[10] Maleki-Ghaleh H., Hossein Siadati M., Fallah A., Zarrabi A., Afghah F., Koc B., Dalir Abdolahinia E., Omidi Y., Barar J., Akbari-Fakhrabadi A. (2021). Effect of Zinc-Doped Hydroxyapatite/Graphene Nanocomposite on the Physicochemical Properties and Osteogenesis Differentiation of 3D-Printed Polycaprolactone Scaffolds for Bone Tissue Engineering. Chem. Eng. J..

[11] Corno M., Rimola A., Bolis V., Ugliengo P. (2010). Hydroxyapatite as a Key Biomaterial: Quantum-Mechanical Simulation of Its Surfaces in Interaction with Biomolecules. Phys. Chem. Chem. Phys..

[12] Vandiver J., Dean D., Patel N., Bonfield W., Ortiz C. (2005). Nanoscale Variation in Surface Charge of Synthetic Hydroxyapatite Detected by Chemically and Spatially Specific High-Resolution Force Spectroscopy. Biomaterials.

[13] Kanzaki N., Onuma K., Ito A., Teraoka K., Tateishi T., Tsutsumi S. (1998). Direct Growth Rate Measurement of Hydroxyapatite Single Crystal by Moire Phase Shift Interferometry. J. Phys. Chem. B.

[14] Hagio T., Tanase T., Akiyama J., Umino M., Iwai K., Asai S. (2009). Difference in Bioactivity, Initial Cell Attachment and Cell Morphology Observed on the Surface of Hydroxyapatite Ceramics with Controlled Orientation. Mater. Trans..

[15] Inagaki M., Kameyama T. (2007). Phase Transformation of Plasma-Sprayed Hydroxyapatite Coating with Preferred Crystalline Orientation. Biomaterials.

[16] Vandecandelaere N., Rey C., Drouet C. (2012). Biomimetic Apatite-Based Biomaterials: On the Critical Impact of Synthesis and Post-Synthesis Parameters. J. Mater. Sci. Mater. Med..

[17] Oyane A., Kawashita M., Nakanishi K., Kokubo T., Minoda M., Miyamoto T., Nakamura T. (2003). Bonelike Apatite Formation on Ethylene-Vinyl Alcohol Copolymer Modified with Silane Coupling Agent and Calcium Silicate Solutions. Biomaterials.

[18] Thein-Han W.W., Misra R.D.K. (2009). Biomimetic Chitosan-Nanohydroxyapatite Composite Scaffolds for Bone Tissue Engineering. Acta Biomater..

[19] Wang Y., Hassan M.S., Gunawan P., Lau R., Wang X., Xu R. (2009). Polyelectrolyte Mediated Formation of Hydroxyapatite Microspheres of Controlled Size and Hierarchical Structure. J. Colloid Interface Sci..

[20] Zhai Y., Cui F.Z. (2006). Recombinant Human-like Collagen Directed Growth of Hydroxyapatite Nanocrystals. J. Cryst. Growth.

[21] Kikuchi M., Ikoma T., Itoh S., Matsumoto H.N., Koyama Y., Takakuda K., Shinomiya K., Tanaka J. (2004). Biomimetic Synthesis of Bone-like Nanocomposites Using the Self-Organization Mechanism of Hydroxyapatite and Collagen. Compos. Sci. Technol..

[22] Venegas S.C., Palacios J.M., Apella M.C., Morando P.J., Blesa M.A. (2006). Calcium Modulates Interactions between Bacteria and Hydroxyapatite. J. Dent. Res..

[23] Dai C., Duan J., Zhang L., Jia G., Zhang C., Zhang J. (2014). Biocompatibility of Defect-Related Luminescent Nanostructured and Microstructured Hydroxyapatite. Biol. Trace Elem. Res..

[24] García-Tuñón E., Couceiro R., Franco J., Saiz E., Guitián F. (2012). Synthesis and Characterisation of Large Chlorapatite Single-Crystals with Controlled Morphology and Surface Roughness. J. Mater. Sci. Mater. Med..

[25] Eslami H., Solati-Hashjin M., Tahriri M. (2009). The Comparison of Powder Characteristics and Physicochemical, Mechanical and Biological Properties between Nanostructure Ceramics of Hydroxyapatite and Fluoridated Hydroxyapatite. Mater. Sci. Eng. C.

[26] Lehmann G., Cacciotti I., Palmero P., Montanaro L., Bianco A., Campagnolo L., Camaioni A. (2012). Differentiation of Osteoblast and Osteoclast Precursors on Pure and Silicon-Substituted Synthesized Hydroxyapatites. Biomed. Mater..

[27] Balamurugan A., Rebelo A.H.S., Lemos A.F., Rocha J.H.G., Ventura J.M.G., Ferreira J.M.F. (2008). Suitability Evaluation of Sol-Gel Derived Si-Substituted Hydroxyapatite for Dental and Maxillofacial Applications through in Vitro Osteoblasts Response. Dent. Mater..

[28] Grandjean-Laquerriere A., Laquerriere P., Jallot E., Nedelec J.M., Guenounou M., Laurent-Maquin D., Phillips T.M. (2006). Influence of the Zinc Concentration of Sol-Gel Derived Zinc Substituted Hydroxyapatite on Cytokine Production by Human Monocytes in Vitro. Biomaterials.

[29] Capuccini C., Torricelli P., Sima F., Boanini E., Ristoscu C., Bracci B., Socol G., Fini M., Mihailescu I.N., Bigi A. (2008). Strontium-Substituted Hydroxyapatite Coatings Synthesized by Pulsed-Laser Deposition: In Vitro Osteoblast and Osteoclast Response. Acta Biomater..

[30] Kannan S., Vieira S.I., Olhero S.M., Torres P.M.C., Pina S., Da Cruz E Silva O.A.B., Ferreira J.M.F. (2011). Synthesis, Mechanical and Biological Characterization of Ionic Doped Carbonated Hydroxyapatite/β-Tricalcium Phosphate Mixtures. Acta Biomater..

[31] Tredwin C.J., Young A.M., Abou Neel E.A., Georgiou G., Knowles J.C. (2014). Hydroxyapatite, Fluor-Hydroxyapatite and Fluorapatite Produced via the Sol-Gel Method: Dissolution Behaviour and Biological Properties after Crystallisation. J. Mater. Sci. Mater. Med..

[32] Cheng K., Weng W., Wang H., Zhang S. (2005). In Vitro Behavior of Osteoblast-like Cells on Fluoridated Hydroxyapatite Coatings. Biomaterials.

[33] Vennat E., Denis M., David B., Attal J.P. (2015). A Natural Biomimetic Porous Medium Mimicking Hypomineralized Enamel. Dent. Mater..

[34] Paine M.L., Snead M.L. (2005). Tooth Developmental Biology: Disruptions to Enamel-Matrix Assembly and Its Impact on Biomineralization. Orthod. Craniofacial Res..

[35] Sibilla P., Sereni A., Aguiari G., Banzi M., Manzati E., Mischiati C., Trombelli L., Del Senno L. (2006). Effects of a Hydroxyapatite-Based Biomaterial on Gene Expression in Osteoblast-like Cells. J. Dent. Res..

[36] Alshemary A.Z., Goh Y.F., Akram M., Razali I.R., Abdul Kadir M.R., Hussain R. (2013). Microwave Assisted Synthesis of Nano Sized Sulphate Doped Hydroxyapatite. Mater. Res. Bull..

[37] Pan Y., Fleet M.E. (2019). Compositions of the Apatite-Group Minerals: Substitution Mechanisms and Controlling Factors. Phosphates Geochem. Geobiol. Mater. Importance.

[38] Koutsopoulos S. (2002). Synthesis and Characterization of Hydroxyapatite Crystals: A Review Study on the Analytical Methods. J. Biomed. Mater. Res..

[39] Fleet M.E., Liu X. (2007). Coupled Substitution of Type A and B Carbonate in Sodium-Bearing Apatite. Biomaterials.

[40] Fleet M.E. (2009). Infrared Spectra of Carbonate Apatites: Ν2-Region Bands. Biomaterials.

[41] Astala R., Stott M.J. (2008). First-Principles Study of Hydroxyapatite Surfaces and Water Adsorption. Phys. Rev. B.

[42] Pastero L., Bruno M., Aquilano D. (2017). About the Genetic Mechanisms of Apatites: A Survey on the Methodological Approaches. Minerals.

[43] Harding I.S., Rashid N., Hing K.A. (2005). Surface Charge and the Effect of Excess Calcium Ions on the Hydroxyapatite Surface. Biomaterials.

[44] Hunter G.K., O’Young J., Grohe B., Karttunen M., Goldberg H.A. (2010). The Flexible Polyelectrolyte Hypothesis of Protein−Biomineral Interaction. Langmuir.

[45] Dubiel E.A., Martin Y., Vermette P. (2011). Bridging the Gap between Physicochemistry and Interpretation Prevalent in Cell−Surface Interactions. Chem. Rev..

[46] Zelzer M., Albutt D., Alexander M.R., Russell N.A. (2012). The Role of Albumin and Fibronectin in the Adhesion of Fibroblasts to Plasma Polymer Surfaces. Plasma Processes Polym..

[47] Liu Z.-S., Tang S.-L., Ai Z.-L. (2003). Effects of Hydroxyapatite Nanoparticles on Proliferation and Apoptosis of Human Hepatoma BEL-7402 Cells. World J. Gastroenterol..

[48] Barbier O., Arreola-Mendoza L., Del Razo L.M. (2010). Molecular Mechanisms of Fluoride Toxicity. Chem.-Biol. Interact..

[49] Eliaz N., Sridhar T.M. (2008). Electrocrystallization of Hydroxyapatite and Its Dependence on Solution Conditions. Cryst. Growth Des..

[50] Faghihi S., Azari F., Szpunar J.A., Vali H., Tabrizian M. (2009). Titanium Crystal Orientation as a Tool for the Improved and Regulated Cell Attachment. J. Biomed. Mater. Res. Part A.

[51] Keikhosravani P., Maleki-Ghaleh H., Kahaie Khosrowshahi A., Bodaghi M., Dargahi Z., Kavanlouei M., Khademi-Azandehi P., Fallah A., Beygi-Khosrowshahi Y., Siadati M.H. (2021). Bioactivity and Antibacterial Behaviors of Nanostructured Lithium-Doped Hydroxyapatite for Bone Scaffold Application. Int. J. Mol. Sci..

[52] Borkowski L., Przekora A., Belcarz A., Palka K., Jojczuk M., Lukasiewicz P., Nogalski A., Ginalska G. (2021). Highly Porous Fluorapatite/β-1,3-Glucan Composite for Bone Tissue Regeneration: Characterization and In-Vitro Assessment of Biomedical Potential. Int. J. Mol. Sci..

[53] Buchloh S., Stieger B., Meier P.J., Gauckler L. (2003). Hepatocyte Performance on Different Crystallographic Faces of Rutile. Biomaterials.

[54] Suzuki K., Fukasawa J., Miura M., Lim P.N., Honda M., Matsuura T., Aizawa M. (2021). Influence of Culture Period on Osteoblast Differentiation of Tissue-Engineered Bone Constructed by Apatite-Fiber Scaffolds Using Radial-Flow Bioreactor. Int. J. Mol. Sci..

[55] Olivier F., Bonnamy S., Rochet N., Drouet C. (2021). Activated Carbon Fiber Cloth/Biomimetic Apatite: A Dual Drug Delivery System. Int. J. Mol. Sci..

[56] Altomare A., Cascarano G., Giacovazzo C., Guagliardi A., Burla M.C., Polidori G., Camalli M. (1994). IUCr SIR92—A Program for Automatic Solution of Crystal Structures by Direct Methods. J. Appl. Crystallogr..

[57] Sheldrick G.M. (2007). A Short History of SHELX. Acta Cryst..

[58] Farrugia L.J. (1997). ORTEP-3 for Windows—A Version of ORTEP-III with a Graphical User Interface (GUI). J. Appl. Crystallogr..

[59] Kotobuki N., Ioku K., Kawagoe D., Fujimori H., Goto S., Ohgushi H. (2005). Observation of Osteogenic Differentiation Cascade of Living Mesenchymal Stem Cells on Transparent Hydroxyapatite Ceramics. Biomaterials.

[60] Chou Y.F., Huang W., Dunn J.C.Y., Miller T.A., Wu B.M. (2005). The Effect of Biomimetic Apatite Structure on Osteoblast Viability, Proliferation, and Gene Expression. Biomaterials.

[61] Pasko P., Bukowska-Strakova K., Gdula-Argasinska J., Tyszka-Czochara M. (2013). Rutabaga (*Brassica Napus* L. Var. Napobrassica) Seeds, Roots, and Sprouts: A Novel Kind of Food with Antioxidant Properties and Proapoptotic Potential in Hep G2 Hepatoma Cell Line. J. Med. Food.

[62] Simmer J.P., Fincham A.G. (1995). Molecular Mechanisms of Dental Enamel Formation. Crit. Rev. Oral Biol. Med..

[63] Cazalbou S., Combes C., Eichert D., Rey C. (2004). Adaptative physico-chemistry of bio-related calcium phosphates. J. Mater. Chem..

[64] Chaikina M.V., Aman S. (2005). Fracture, Grinding, Mechanical Activation and Synthesis Processes in Solids under Mechanical Action. Sci. Sinter..

[65] Henderson C.E. (2011). Protocols and Pitfalls of Electron Microprobe Analysis of Apatite. Master’s Thesis.

[66] Hahn T., International Union of Crystallography (2005). International Tables for Crystallography, Volume A: Space-Group Symmetry.

